# Exoskeleton may influence the internal body temperatures of Neotropical dung beetles (Col. Scarabaeinae)

**DOI:** 10.7717/peerj.3349

**Published:** 2017-05-18

**Authors:** Valentina Amore, Malva I.M. Hernández, Luis M. Carrascal, Jorge M. Lobo

**Affiliations:** 1 Departamento de Ecologia e Zoologia, Universidade Federal de Santa Catarina, Florianópolis, Santa Catarina, Brazil; 2 Department of Biogeography and Global Change, Museo Nacional de Ciencias Naturales, CSIC, Madrid, Spain

**Keywords:** Cuticle properties, Body size, Heating process, Passive physiology, Interspecific differences

## Abstract

The insect exoskeleton is a multifunctional coat with a continuum of mechanical and structural properties constituting the barrier between electromagnetic waves and the internal body parts. This paper examines the ability of beetle exoskeleton to regulate internal body temperature considering its thermal permeability or isolation to simulated solar irradiance and infrared radiation. Seven Neotropical species of dung beetles (Coleoptera, Scarabaeinae) differing in colour, surface sculptures, size, sexual dimorphism, period of activity, guild category and altitudinal distribution were studied. Specimens were repeatedly subjected to heating trials under simulated solar irradiance and infrared radiation using a halogen neodymium bulb light with a balanced daylight spectrum and a ceramic infrared heat emitter. The volume of exoskeleton and its weight per volume unit were significantly more important for the heating rate at the beginning of the heating process than for the asymptotic maximum temperature reached at the end of the trials: larger beetles with relatively thicker exoskeletons heated more slowly. The source of radiation greatly influences the asymptotic temperature reached, but has a negligible effect in determining the rate of heat gain by beetles: they reached higher temperatures under artificial sunlight than under infrared radiation. Interspecific differences were negligible in the heating rate but had a large magnitude effect on the asymptotic temperature, only detectable under simulated sun irradiance. The fact that sun irradiance is differentially absorbed dorsally and transformed into heat among species opens the possibility that differences in dorsal exoskeleton would facilitate the heat gain under restrictive environmental temperatures below the preferred ones. The findings provided by this study support the important role played by the exoskeleton in the heating process of beetles, a cuticle able to act passively in the thermal control of body temperature without implying energetic costs and metabolic changes.

## Introduction

It is assumed that the appearance of an external cuticle rich in chitin was, on the basis of the radiation of arthropods, facilitating the insect colonization of terrestrial environments (Brusca & Brusca, 2003; Minelli, Boxshall & Fusco, 2013). Insect cuticle is an extracellular layer covering the outer surface of the animal that rests on a layer of epidermal cells. Basically, it is organized in two structurally distinct horizontal layers: the thin epicuticle composed of lipids, waxes and proteins able to protect the animal against dehydration and soaking (Gibbs, 2011), and the thicker and inner procuticle, the principal component of which is chitin. The insect exoskeleton represents a multifunctional coat exhibiting a continuum of mechanical and structural properties that are tailored to needs and functions (Hillerton, Reynolds & Vincent, 1982), and has inspired research especially in the area of bio-materials. Just to mention a few exoskeleton properties: it defines shape and color of the body, and therefore crypsis, aposematism, mimicry, sexual and visual signaling; it enables attachment of muscles and organs; it constitutes a physical barrier between the body’s inside and outside regulating air and water retention; it allows food grinding and filtering, body cleaning and lubrication, adhesion or sound generation (Ifuku & Saimoto, 2012; Fernandez & Ingber, 2013).

Some studies also suggest the implication of the cuticle in the thermal response of insects (Vincent & Wegst, 2004). Reticulation changes (Drotz, Brodin & Nilsson, 2010), thin patches (Slifer, 1953) or color variation (Trullas, Van Wyk & Spotila, 2007; Gross, Schmolz & Hilker, 2004; Mikhailov, 2001; Schweiger & Beierkuhnlein, 2016) have been considered capable to be adaptive in controlling the thermal response of insects. However, a large number of the studies aimed at examining the temperature acquisition and regulation in insects traditionally considered active physiological adaptations and behavioral strategies (Heinrich, 1974). This is the case, for example, of desert Hemiptera of the Cicadidae family (Sanborn, Heath & Heath, 1992), Coleoptera of the families Cetoniidae (Saeki, Kruse & Switzer, 2005) or Scarabaeidae (Verdú, Diaz & Galante, 2004; Verdú, Arellano & Numa, 2006), and Dipterans and Hymenopterans pollinators (Herrera, 1997).

**Figure 1:**
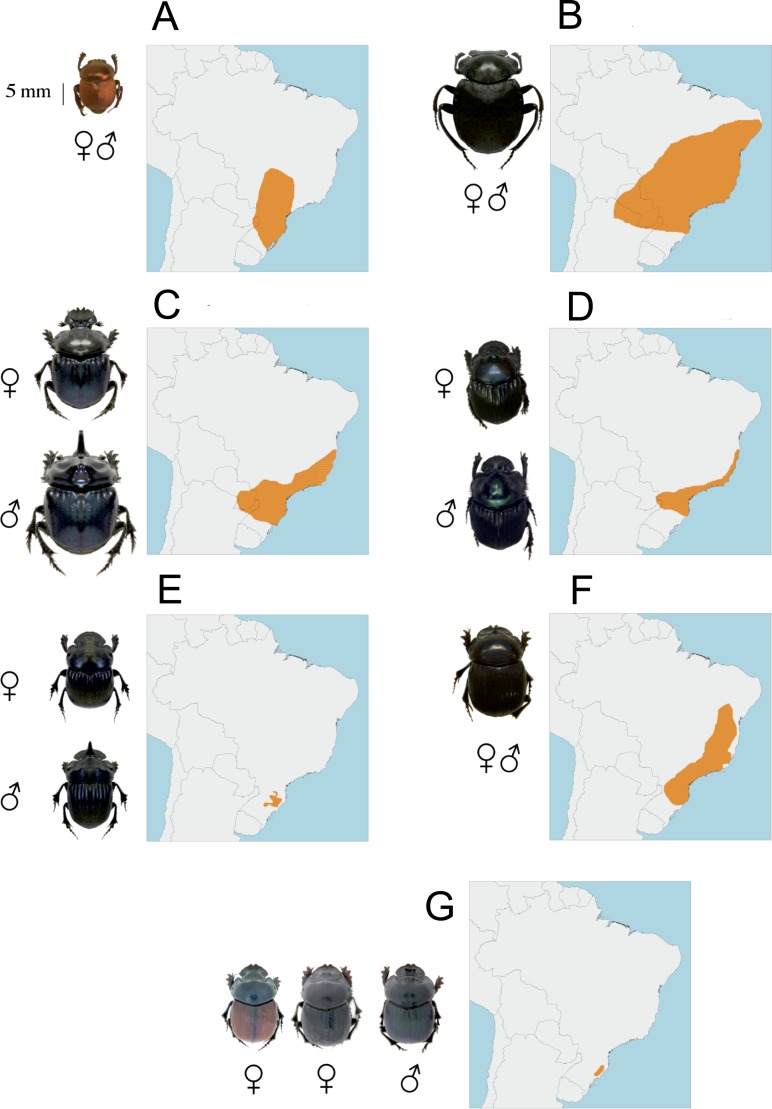
Dorsal habitus of *Canthon rutilans rutilans* (A), *Deltochilum brasiliense* (B), *Coprophanaeus saphirinus* (C), *Phanaeus splendidulus* (D), *Homocopris* sp. nov (E), *Dichotomius sericeus* (F) *Dichotomius opalescens* (G) showing their sexual dimorphism as well as main within species coloration variations. The maps represent the general distribution of each species.

The primary heating source for life on our planet comes from the sun (Wild et al., 2005). Solar radiation is constituted by electromagnetic waves that penetrate the atmospheric window (mainly from 290 to 2,600 nm) belonging to ultraviolet (UV), visible and infrared radiations which constitute the main sources of the heat gained by living forms (Tracy, 1979). Considering that the insect exoskeleton constitutes the barrier between these electromagnetic waves and the internal body parts, we aim in this study to examine the ability of beetle exoskeleton to regulate internal body temperature, a topic that has rarely been studied (Carrascal, Jiménez-Ruiz & Lobo, 2017). The thermal permeability or isolation of the exoskeleton to two radiation sources is studied in seven Neotropical species of dung beetles (Coleoptera, Scarabaeinae; Fig. 1) differing in color, surface sculptures, size, sexual dimorphism, period of activity, guild category and altitudinal distribution in order to examine whether there are interspecific differences. These species were selected because they belong to a diversified beetle group characterized by the consumption of an ephemeral resource requiring a high flight performance, and also because they spend part of their activities exposed to infrared radiation (i.e., when they are inside tunnels in the ground) and part exposed to solar irradiance, (i.e., when they walk or fly looking for dung).

## Material and Methods

### Experimental conditions and design

A variable number of individuals of seven Neotropical Scarabaeinae species were studied (Table 1). These species belong to six genera which differ widely both in their morphological and ecogeographical characteristics (Table 1; Fig. 1), thus maximizing the possibility of finding interspecific differences. The specimens belong to a series of 228 individuals collected between June of 2015 and June of 2016 in four sites between 800 and 1600 m a.s.l in Atlantic Forest formations of the Santa Catarina state, southern Brazil. All these individuals were preserved in alcohol as part of the entomological collection of the department of Ecology and Zoology of the Federal University of Santa Catarina. Each specimen was dried during at least 48 h in a stove at 50 °C. The selection of the individuals of each species was carried out looking for a similar representation of males and females, especially for those species that show sexual dimorphism (see Table 1; Fig. 1). The taxonomic identification of these species is based on the entomological collections of the Universidade Federal de Santa Catarina and the Universidade Federal de Mato Grosso (Brazil) which are supervised by Fernando Zagury Vaz-de-Mello. The permission to collect dung beetles were issued by Instituto Chico Mendes de Conservação da Biodiversidade (ICMBio/MMA, permit # 49486-1), the Brazilian regulatory institution on biodiversity. Only one of the selected species remains unidentified (*Homocopris* sp. nov.) because it belongs to a genus recently ratified (Vaz-de Mello, Génier & Smith, 2010) and under a review process, which approximately may contain eight species (Silva, Vaz-de Mello & Di Mare, 2011). According to the taxonomic authority of this genus (Fernando Zagury Vaz-de-Mello) all the collected specimens of *Homocopris* correspond to the same species, which will be described in the near future.

**Table 1:** List of studied Scarabaeinae species where the number of individuals analyzed for each species is specified together with other biological features such as pseudo-volume (cm^3^ ± sd), dry mass (g ± sd), exoskeleton color, activity period (D, diurnal; C, crepuscular; N, nocturnal) guild (P, paracoprid; T, telecoprid), presence of sexual dimorphism and altitudinal distribution.

Species	*N*	Pseudo- volume (cm^3^)	Dry mass (mg)	Color	Activity	Guild	Sexual dimorphism	Altitudinal distribution
*Deltochilum brasiliense* Castelnau, 1840	16 (9 ♀, 7 ♂)	1.60 ± 0.32	411.9 ± 155.1	Black	N	T	No	Medium- highland
*Canthon rutilans rutilans* Harold, 1868	20 (10 ♀, 10 ♂)	0.23 ± 0.05	72.2 ± 28.1	Red	D	T	No	Lowland
*Coprophanaeus saphirinus* (Sturm, 1857)	18 (9 ♀, 9 ♂)	1.13 ± 0.28	293.7 ± 98.3	Dark-blue	C	P	Yes	Lowland
*Phanaeus splendidulus* (Fabricius, 1781)	20 (10 ♀, 10 ♂)	0.84 ± 0.16	271.4 ± 50.2	Dark-blue	C	P	Yes	Lowland
*Homocopris sp. nov.*	17 (8 ♀, 9 ♂)	0.66 ± 0.12	193.0 ± 35.0	Black	N	P	Yes	Medium- highland
*Dichotomius sericeus* Harold, 1867	15 (9 ♀, 6 ♂)	0.45 ± 0.09	99.6 ± 30.6	Black	N	P	No	Lowland
*Dichotomius opalescens* Felsche, 1910	15 (7 ♀, 8 ♂)	0.35 ± 0.06	101.6 ± 21.4	Dark-blue -brown	N	P	Yes	Highland
Total	121 (62 ♀, 59 ♂)							

Thorax length (*l*), width (*w*) and height (*h*) were measured for each specimen with a manual millimeter gauge in order to calculate the pseudo-volume (*V*) of each specimen considering the volume of an ellipsoid and converting the linear dimensions in approximate radius ($V= \frac{\pi }{3} l \left( \frac{w}{2} \right) h$). The body dry mass of each specimen was also measured by using a scale with a precision of ±0.0001 g. The weight of chitin per volume unit (hereafter ChW) was calculated for all specimens by linearly regressing dry mass on pseudo-volume and retaining the thus generated regression residuals. The allometric relationship between these two variables was obtained by log-transforming the data of both variables (*R*^2^ = 0.833, *n* = 121, slope = 0.94). As a result, ChW is the weight of each specimen relative to its volume, both measurements being orthogonal (i.e., completely independent, *r* = 0).

Each individual was pierced in the apex of the pygidium with an entomological pin in order to insert a type-K thermocouple (Chromel/Alumel) inside the exoskeleton, reaching the middle of the abdominal cavity avoiding the direct contact with the cuticle. The thermocouple wire (0.3 mm Ø) was welded at the pygidium using a small drop of hot melt silicone applied with an electric glue gun, and it was plugged into a digital thermometer (4 channels thermometer, type K/J/R/E/T/S; Pt100ohm OMEGA RDXL4SD) used for recording body internal temperatures. Before each trial, the specimen with the welded wire was placed in a freezer until reaching a temperature of around −20 °C. Such a low temperature allowed time to place the individual in the right position under the radiation source and so start the experiments at a temperature of ca. 0 °C (time 0). Our experiments aimed at measuring the direct influence of two different radiation sources (simulated solar irradiance and infrared radiation; SR and IR, respectively) on the internal body temperature of the studied specimens. The dorsal part of each individual was exposed to SR and the ventral part to IR assuming that in nature the upper part of the exoskeleton principally interacts with solar radiance, while the ventral part will be mainly exposed to infrared radiation such as that which comes from the soil. SR was simulated by using a 100 watt halogen neodymium bulb light with a balanced daylight spectrum (Exo Terra Sun-Glo Daylight Halogen Lamp 100 watt), while IR was generated by a ceramic infrared heat emitter of 2,000 watt (pyroceramic digital heating plate; HIPPER QUIMICA). Each specimen was placed five cm over the experimental arena suspended on the thermocouple wire by means of a clamp, without coming into contact with any surface. For each experimental trial, the dorsal part of each individual was firstly exposed to SR. Subsequently, after a new cooling down period, the ventral part was exposed to IR. The SR source radiated perpendicularly to the dorsal area of the experimental specimen, and the IR source radiated perpendicularly to the ventral area of the beetle. One thermocouple was always located 3 cm to the right of each specimen in order to control the random, subtle, temperature variations surrounding each individual during the trials (T_cont_). The control temperature (T_cont_) integrates all heat sources from light and IR bulbs, including the temperature of the surrounding air. The metal tip of the thermocouple was directly irradiated by the bulbs, so it was affected by the source of radiation. This control temperature was used as a covariate to estimate and correct heating parameters in the models. The mean value (±95% confidence interval) of T_cont_ during the trials was 27.1 ±0.1°C. The radiation emitters were located at a distance from each specimen in order to attain the same control T_cont_ throughout all assays (7 cm for IR and ca. 35 cm for SR).

Internal body temperature was recorded each second during 10 min, time enough to reach a stable temperature (asymptotic value, see below). Temperature measurements were carried out by means of a Fluke 54 II B dual digital thermometer (0.05% accuracy). In order to obtain stabilized and comparative measurements these temperature records were repeated three times for each specimen and source of radiation (SR or IR), and the obtained average temperatures subsequently adjusted to an exponential growth function [temperature =a * (*b* –*exp* (−*c* * time))], using Curve Expert 1.4 for Windows. The *c* parameter represents the heating rate of the internal body temperature at the beginning of the heating process, while the product *a* *b is the asymptote or the maximum temperature finally reached by the body of each specimen. Within-individual variation in heating parameters across trials is very low in these essays, accounting for a very small percentage of variance (less than 5%; Carrascal, Jiménez-Ruiz & Lobo, 2017). In order to estimate the variability in temperature measurements due to differences in the piercing of the thermocouple in the apex of the pygidium, a prior essay was done extracting and re-entering the thermocouple 10 times in the same individual under simulated solar irradiance conditions. This pilot study was carried out with the smaller species (*Canthon rutilans rutilans*) in order to increase the possibility of variability in temperature measurements. The coefficient of variation (standard deviation/mean × 100) of the asymptotic maximum temperature was 1.29, while the coefficient of variation of the heating at the beginning of the trials was 7.85.

### Data analyses

In total, 726 thermal data sets were obtained (121 individuals of seven species × 2 radiation sources × 3 repetition measurements) with a nearly equal representation of individuals among species (see Table 1). The whole variation in the rate of heat gain at the beginning of the heating trials (heating rate, *c*) and the maximum temperature reached (asymptote of the temperature gain curve, a*b) were analyzed by means of three-way ANCOVAs using a type III sum of squares (i.e., estimating the partial effects of each factor while controlling for the effects of the remaining predictors). Species identity (seven taxa), sex (two levels) and the radiation source (SR and IR) were the factors, while the animal pseudo-volume (in logarithm), the chitin weight per volume unit (ChW), and the control temperature (T_cont_) during the trials were the covariates. Recall that T_cont_ is not really a predictor of interest, but a covariate introduced into the model in order to cope with minor, random, variations in temperature. Model residuals were checked for normality and homoscedasticity assumptions. Due to deviations from homoscedasticity of the residuals across the predictions of the models (mainly for the asymptote), we used the heteroscedasticity-corrected coefficient covariance matrix in order to obtain the significance of the effects in the model (Zeileis, 2004); the HC4 estimator suggested by Cribari-Neto (2004) was used to further improve the performance in significance estimations, especially in the presence of influential observations under small sample sizes. In order to avoid the wrong interpretation of main effects when significant interaction terms between factors were detected (i.e., type IV error; Marascuilo & Levin, 1970) we carried out post-hoc analyses of interactions in the factorial designs. To overcome the inflation of type I error derived from performing multiple related tests, we applied Holm’s adjustment, a widely recognized approach designed to give strong control of the family-wise type I error rate. Average figures for each heating parameter in the interaction *taxa* *sex *source of radiation were adjusted after taking into account the influence of the covariates in the models.

The robustness and repeatability of the results of the ANCOVA models were tested by means of five-fold cross-validations, repeated ten times for both the asymptote and the heat gain at the beginning of the heating trials. The average correlation between predictions and observed figures in the response variables were very high in both ANCOVA models (asymptote: *r* = 0.829, *sd* = 0.008; heating rate: *r* = 0.871, *sd* = 0.007), showing no bias (i.e., ***a*** was approximately zero and ***b*** was approximately one in the equation *observed* = ***a*** + b*predicted).

In order to obtain robust inferences from our experimental design, we increased the type I error rate to 0.005, as the critical reference level for significance, following Johnson (2013) and Colquhoun (2014). All analyses were carried out under R version 3.1.2 (R core Team, 2014), using the packages *car*, *phia*, *sandwich* and *DAAG*.

## Results

**Figure 2:**
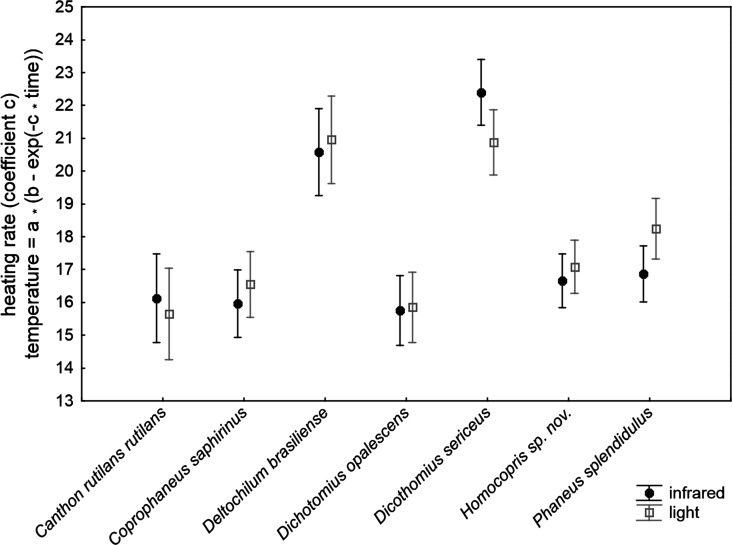
Mean ± one standard error of heating rates at the beginning of the heating trials in seven dung beetles (Scarabaeinae) under two sources of radiation: infrared radiation (infrared) and luminous radiation (light) resembling the electromagnetic spectrum of sun radiation. The values presented are adjusted means after controlling for the effect of the three covariates included in the ANCOVA model of Table 3 (control temperature, animal volume and weight per volume unit).

The ANCOVA model with the increasing rate of body temperature at the beginning of the heating experiments (coefficient *c* of the exponential function relating temperature to time) was highly significant (*F*_30,211_ = 32.42, *P* < 0.001; *R*^2^ = 0.822; Table 2). The subtle random variations in the control temperature among trials did not significantly affect the outcomes of the experiment. The volume and chitin weight per volume unit (ChW) of beetle exoskeletons had a negative influence and a prominent role on heating rate both on the grounds of significance and effect size (see P and partial-eta^2^ in Table 3). Volume explained 46% of variance by itself and ChW 29%: larger beetles with apparently thicker exoskeletons heated more slowly. There were highly significant differences of low magnitude among species after controlling for these three covariates (5.3% of total variance accounted for). The source of radiation did not affect the heating rate. Figure 2 shows the interspecific variation in heating rate under both sources of radiation. *Canthon rutilans rutilans*, *Coprophanaeus saphirinus*, *Dichotomius opalescens*, *Homocopris* sp. nov. and *Phanaeus splendidulus* were the species with the lowest heating rates, while *Dichotomius sericeus* and *Deltochilum brasiliense* had the highest rates (see also Table 2). There were no global differences among sexes in the heating rates, although there was a significant interaction term *taxa* *sex (*P* = 0.009) with a partial effect that accounted for a very low proportion of total variance (0.6%); it was mainly attributable to intersexual differences in *Dicothomius sericeus*, with females heating slightly faster than males (see Table 3). The remaining interaction terms were not significant.

**Table 2:** Means and standard error (se) of heating rate (c*1,000; *c* in °C/s) and maximum temperature (asymptote; °C) during heating trials under laboratory conditions by seven taxa of dung beetles (Scarabaeinae) according to sex and two sources of radiation. Figures are adjusted means after controlling for the influence of three covariates in the ANCOVA models of Tables 3 and 4. The coefficient *c* has been multiplied by 1,000 in order to gain significant digits. IR: infrared radiation. SR: luminous radiation resembling the electromagnetic spectrum of sun radiation. *N*: number of different used individual beetles. All data refer to the internal temperature of dried specimens.

Taxa	Sex	*n*	Source	c*1,000	se	Asymptote	se
*Canthon rutilans rutilans*	f	10	IR	16.3	1.43	27.4	0.96
*Canthon rutilans rutilans*	f	10	SR	16.8	1.45	31.0	0.97
*Canthon rutilans rutilans*	m	10	IR	15.8	1.52	27.4	1.01
*Canthon rutilans rutilans*	m	10	SR	14.4	1.56	32.7	1.04
*Coprophaneus saphirinus*	f	9	IR	16.3	1.30	28.2	0.87
*Coprophaneus saphirinus*	f	9	SR	17.0	1.27	32.3	0.85
*Coprophaneus saphirinus*	m	9	IR	15.5	1.17	28.7	0.78
*Coprophaneus saphirinus*	m	9	SR	16.1	1.16	32.0	0.78
*Deltochilum brasiliense*	f	9	IR	21.6	1.45	29.1	0.97
*Deltochilum brasiliense*	f	9	SR	21.8	1.45	35.1	0.97
*Deltochilum brasiliense*	m	7	IR	19.1	1.53	28.6	1.03
*Deltochilum brasiliense*	m	7	SR	19.6	1.53	33.9	1.03
*Dichotomius opalescens*	f	7	IR	15.3	1.32	27.7	0.88
*Dichotomius opalescens*	f	7	SR	16.1	1.32	32.9	0.88
*Dichotomius opalescens*	m	8	IR	17.0	1.30	27.2	0.87
*Dichotomius opalescens*	m	8	SR	16.5	1.29	33.8	0.86
*Dichotomius sericeus*	f	9	IR	21.7	1.09	27.6	0.73
*Dichotomius sericeus*	f	9	SR	19.9	1.10	30.3	0.74
*Dichotomius sericeus*	m	6	IR	22.7	1.43	28.2	0.96
*Dichotomius sericeus*	m	6	SR	21.6	1.42	32.5	0.95
*Homocopris sp. nov*	f	8	IR	16.2	1.14	28.1	0.76
*Homocopris sp. nov*	f	8	SR	16.2	1.11	30.7	0.74
*Homocopris sp. nov*	m	9	IR	17.8	1.07	28.0	0.71
*Homocopris sp. nov*	m	9	SR	18.7	1.08	33.0	0.72
*Phanaeus splendidulus*	f	10	IR	16.4	1.07	30.1	0.72
*Phanaeus splendidulus*	f	10	SR	18.8	1.07	42.7	0.72
*Phanaeus splendidulus*	m	10	IR	16.8	1.08	29.4	0.72
*Phanaeus splendidulus*	m	10	SR	16.9	1.22	36.1	0.81

**Table 3:** Results of the ANCOVA model examining the influence of three covariates (subtle random variation of temperature during trials, animal volume and weigh per volume unit) and three factors (seven species, two sexes and two sources of radiation) on the heating rate at the beginning of the heating experiments under laboratory conditions (measured internally in beetle exoskeletons). Beta: standardized regression coefficients for the effect of covariates on the response variable. Partial-eta^2^: effect size according to the ratio SS_*effect*_/(SS_*effect*_ + SS_*error*_).

	Beta	Partial-eta^2^	df	F	*P*
Control temperature	0.048	0.007	1, 211	1.61	0.206
Animal volume (log)	−0.742	0.200	1, 211	53.43	<0.001
Weight per volume unit	−0.500	0.495	1, 211	88.03	<0.001
Source of radiation		0.005	1, 211	0.12	0.732
Taxa		0.256	6, 211	11.69	<0.001
Sex		0.006	1, 211	0.13	0.720
Source * Taxa		0.018	6, 211	0.45	0.844
Source * Sex		0.002	1, 211	0.44	0.510
Taxa * Sex		0.058	6, 211	2.93	0.009
Source * Taxa * Sex		0.010	6, 211	0.48	0.826

**Table 4:** Results of the ANCOVA model examining the influence of three covariates (subtle random variation of temperature during trials, animal volume and weight per volume unit) and three factors (seven species, two sexes and two sources of radiation) on the maximum temperature internally reached by beetle exoskeletons of seven species in heating experiments (asymptotic temperature attained after 10 min). Beta: standardized regression coefficients for the effect of covariates on the response variable. Partial-eta^2^: effect size according to the ratio SS_*effect*_/(SS_*effect*_ + SS_*error*_).

	Beta	Partial-eta^2^	df	F	*P*
Control temperature	0.066	0.010	1, 211	2.30	0.131
Animal volume (log)	−0.017	0.000	1, 211	0.02	0.899
Weight per volume unit	0.043	0.006	1, 211	0.81	0.369
Source of radiation		0.631	1, 211	386.01	<0.001
Taxa		0.346	6, 211	11.95	<0.001
Sex		0.001	1, 211	0.16	0.691
Source * Taxa		0.190	6, 211	5.12	<0.001
Source * Sex		0.000	1, 211	0.02	0.899
Taxa * Sex		0.151	6, 211	4.28	<0.001
Source * Taxa * Sex		0.104	6, 211	3.14	0.006

**Figure 3:**
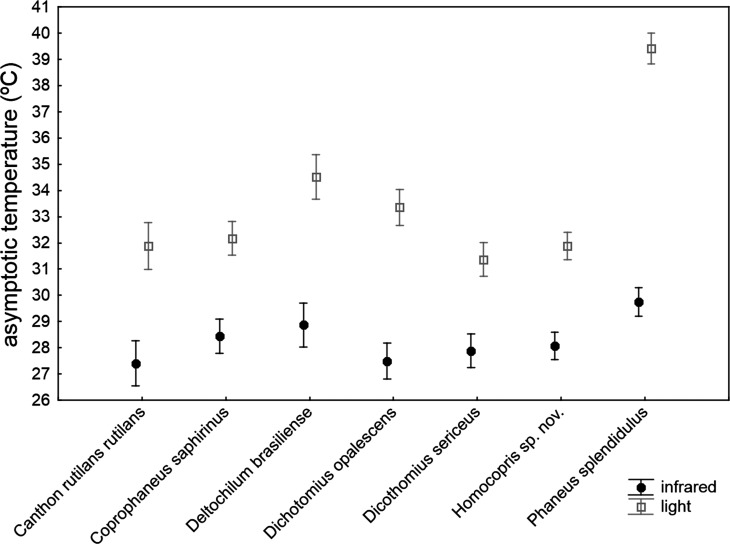
Mean ± one standard error of the maximum temperature internally reached by beetle exoskeletons in heating experiments (asymptotic temperature attained after ten minutes) in seven dung beetles (Scarabaeinae) under two sources of radiation: infrared radiation (infrared) and luminous radiation (light) resembling the electromagnetic spectrum of sun radiation. The values presented are adjusted means after controlling for the effect of the three covariates included in the ANCOVA model of Table 4 (control temperature, animal volume and weight per volume unit).

The asymptotic maximum temperature reached at the end of heating trials was significantly explained by the ANCOVA model (Table 4): *F*_30,211_ = 23.24, *P* < 0.001; *R*^2^ = 0.768. The three covariates (subtle stochastic variations among trials in control temperature, animal volume and ChW) had negligible effects on the measured asymptotic body temperatures (*P* > 0.14), with very low partial magnitude effects (partial-eta^2^ < 0.01; the three covariates accounted for 1.1% of total variance). There were highly significant differences among species that accounted for 18.9% of total variance, with the second highest partial magnitude effect. The source of radiation was the predictor with the largest effect size (partial-eta^2^ = 0.63), showing a high significant influence (*P* < 0.001) and accounting for 41.8% of total variance: beetle exoskeleton attained higher temperature (ca. 5 °C) under artificial sunlight than under infrared radiation. Nevertheless, this pattern was not homogeneously generalizable across taxa (see the significance of the interaction term source*taxa in Table 4; *P* < 0.001). Thus, the *a posteriori* test comparing the seven species in the infrared trials showed no statistical differences among species (*F*_6,211_ = 1.47, *P* = 0.19 with Holm adjustment), while there was a highly significant difference in the asymptotic temperature among species using artificial sunlight as the source of radiation (*F*_6,211_ = 23.79, *P* < 0.001 with Holm adjustment). Therefore, although there were important differences among species in the maximum temperature finally reached in heating trials, the magnitude of these differences were negligible under infrared radiation, but were very prominent under simulated sunlight radiation, with the highest values in *Phanaeus splendidulus* and the lowest in *Dichotomius sericeus*, *Homocopris* sp. nov. and *Canthon rutilans rutilans* (see Table 2 and Fig. 3). Globally, there were no significant differences between sexes in the temperature reached after heating trials, although the interaction term *taxa* *sex reached significance (*P* < 0.001) with a low partial magnitude effect and accounting for 4.2% of total variance. The only species responsible of this interaction was *Phanaeus splendidulus* (*P* < 0.001 in the post-hoc test with Holm adjustment): females reached higher asymptotic temperatures than males, mainly under simulated solar radiation (see Table 2). Consistent with the significance of these two interactions terms, the whole interaction *source* *taxa *sex was also significant.

Comparing the figures of partial-eta^2^ obtained in both ANCOVA models, it is possible to ascertain the different magnitude effects attained by the predictor variables in determining the two heating parameters of the beetle exoskeleton. The volume and ChW of exoskeleton were more important for the heating rate at the beginning of the heating process than for the asymptotic maximum temperature reached at the end of the trials. On the other hand, the source of radiation had a major influence on the asymptotic temperature reached, but a negligible effect in determining the rate of heat gain by beetles. Interspecific differences had marginally greater magnitude in the asymptotic temperature. Intersexual differences *per se* had an unimportant influence in both heating parameters, mainly manifesting their influence through the interaction with some taxa (i.e., different intersexual differences across species, mainly restricted to *Phanaeus splendidulus*).

## Discussion

The heating process of internal body parts for poikilothermic animals such as dung beetles is crucial for the completion of daily activities, and life in general (May, 1979). Two main phases can be distinguished when a beetle exoskeleton is submitted to an external source of radiation: a first phase of quick heat gain from cold conditions (measured here by means of the heating rate), and a second phase in which the internal body cavity is reaching gradually the thermal equilibrium (estimated by the asymptote).

The first phase would represent the response of a beetle when it passes from inactivity to activity (e.g., in the early morning for diurnal beetles). In this case, our results indicate that the type/source of radiation does not seem to influence the variation in the heating rates of the different species. This suggests that the initial body warm up of these species would be passively facilitated by the permeability of the exoskeleton to infrared radiation. If this were the case, the source for initial heating would come just from the soil because this is where beetles spend their periods of inactivity (Scholtz, Davis & Kryger, 2009). Interestingly, a similar result was obtained for the Palaearctic species of a different Scarabaeoidea family (Geotrupidae) thus suggesting the general capacity of these beetle exoskeletons to acquire heat from infrared radiations (Carrascal, Jiménez-Ruiz & Lobo, 2017). The ability of the exoskeleton to quickly acquire heat from soil infrared radiation can be an extremely advantageous characteristic for animals such as dung beetles as they spend much of their lives inside tunnels they dig in the ground (Simmons & Ridsdill-Smith, 2011). Unlike what happens in Geotrupidae (see Carrascal, Jiménez-Ruiz & Lobo, 2017) heating rates differ between the considered species, so that *Deltochilum brasiliense* and *Dichotomius sericeus* would have a faster rate of heat acquisition than the remaining considered species. Both species are black coloured and nocturnal dung beetles.

The second phase, the maximum temperature reached by the body (asymptote), may reflect the thermal insulation or permeability of the exoskeleton to different radiation sources in order to facilitate or prevent the heat exchange of individuals. Our results indicate that the source of radiation is important in determining the internal temperature. Remarkably, the internal temperature finally reached does not differ between the considered species when they are ventrally submitted to infrared radiation, but it does for the dorsal exposition to simulated sun irradiance. This implies that both visible and near-UV light is absorbed dorsally by the exoskeleton and transformed in heat, a process that could facilitate the heat gain under environmental temperatures below the preferred ones in many species including both endotherms and ectotherms (e.g., Heatwole & Taylor, 1987; Lustick, Battersby & Kelty, 1978; Lustick, Adam & Hinko, 1980; Belliure & Carrascal, 2002). Thus, our results reveal that probably there are exoskeletons with a differential capacity of transforming the visible and UV light into heat. In a former study carried out with Geotrupidae species (Carrascal, Jiménez-Ruiz & Lobo, 2017) the dorsal exposition to simulated sun irradiance also significantly increase the internal heat of individuals. We hypothesize that the different responses of the species’ exoskeletons to solar-type radiation could contribute to explain the differential daily and seasonal periods of activity of the species or their microhabitat selection. For example, *Phanaeus splendidulus* seem to be a species capable of reaching higher internal temperatures being a diurnal and crepuscular species, living inside the forest where solar radiation is not very intense. In any case, more data are needed prior to rendering a decision on the role played by the thermal capacities of the exoskeleton in environmental preferences.

The temperature finally reached by the body did not appear to be influenced by physical conditions such as body volume and exoskeleton weight per volume unit (but see Herrera, 1997). However, these physical factors strongly influence the heating rate, suggesting that increases or decreases of body size and/or ChW could prevent or facilitate the fast internal heating of the beetle body. A similar result was obtained in the case of Geotrupidae species (Carrascal, Jiménez-Ruiz & Lobo, 2017). The influence of ChW can be due to changes in the density of the exoskeleton, variations in cuticle thickness, and/or a modification in the volume of air sacs and tracheae which facilitate thermoregulation (Ishay et al., 2004; Verdú, Alba-Tercedor & Jiménez-Manrique, 2012). In the absence of more thorough analyses, we suspect that changes in body size and cuticle weight per volume could be important in facilitating a quick response from inactivity to an active state.

As the mass of the exoskeleton isometrically increases with body size (Lease & Wolf, 2010), those species with a larger body size may have a lower capacity for either passive heat gain or loss, being the species more probably showing endogenous thermoregulation. For example, Verdú, Arellano & Numa (2006) found that larger dung beetles (>1.9 g) elevate and maintain their body temperature at levels well above ambient temperature, whereas smaller beetles’ body temperatures conform with ambient temperature. This may imply a trade-off between the energetic efficiency attributed to larger insects (Reim, Teuschl & Blanckenhorn, 2006) and the ability to passively manage internal heating. Moreover, the role played by body mass on heating rates when coming from cold may be the basis for thermal niche partitioning and geographical distribution patterns (Herrera, 1997; Verdú, Arellano & Numa, 2006). Results provided here suggest than this capacity of heat interchange would be mainly due to the management of infrared wavelengths. The insect cuticle might have thermoelectric properties (Ishay et al., 2003) and thinner insect cuticles have been related with faster rates of insecticide absorption in *Anopheles* (Wood et al., 2010), the occurrence of predator running escape responses in cockroaches (Clark & Triblehorn, 2014), higher rates of heat dissipation in bees (Abou-Shaara, 2015) and heat conductivity (Galusko et al., 2005). Thus, although the data are too few to draw any generalizations with confidence, it is clear that the exoskeleton of some species seem to act differentially in the process of heat transfer from external radiation sources in the absence of active physiological functions.

Available studies in coleoptera (Kinoshita, 2008), and specifically in dung beetles (Pye, 2010; Akamine et al., 2011), show that color variations are not pigmentary, but structural. Thus, beetle’s color is understood as a pure physical mechanism due to material properties of the exoskeleton which reflects, scatters and deflects the light. Nevertheless, (Schweiger & Beierkuhnlein, 2016) have recently claimed the thermoregulatory importance of dark coloration in carabid beetles, which in turn strongly depends on species body size. According to that hypothesis, colour should influence heat gain in insects, and thus it is expected to find that darker beetles attain higher heating rates and maximum temperatures than lighter ones when physical conditions remain constant. This is not clearly the case with our studied species according to interspecific variation in color casts shown in Fig. 1 and the results presented in Figs. 2 and 3. For example, *Canthon rutilans* and *Homocopris* sp. nov. have indistinguishable adjusted heating rates and asymptotic temperatures under simulated sun radiation, in spite of the fact that the first species has a light red color cast while the second has a deep black color. On the other hand, two dark species such as *Deltochilum brasiliense* and *Coprophanaeus saphirinus* have very different heating rates (recall that these comparisons are established after controlling for the interspecific differences in animal volume and exoskeleton weight per volume unit). Moreover, interspecific differences in the heating rates are similar across species under the two sources of radiation (*P* = 0.732), although differences in the color cast of the beetle species are expected to have an effect with visible light not paralleled by a similar response under infrared radiation.

More laboratory studies are necessary to assess which, if any, of the morphological (e.g., microsculptures, convexity of the elytra), structural (e.g., epicuticle layers of lipids, waxes and proteins) and color characteristics of the beetle cuticle are candidates to causally explain the interspecific differences in heat management found for the first time in this study. Likewise, more experimental ecological studies are needed to examine the possible correspondence of the environmental, phylogenetic and biogeographical features of the species with the probable role played by the exoskeleton as a passive source of thermal adjustments. Bearing in mind previous considerations, the provided results suggest that this correspondence must be complex. For example, in our small group of dung beetle species, those living under cooler conditions, such as *Homocopris sp*. or *Dichotomius opalescens*, are able to reach internal temperatures and have heating rates similar to those other species living under more tropical conditions, such as *Coprophanaeus saphirinus* or *Dichotomius sericeu*s.

## Conclusions

The findings provided by this study represent a further evidence supporting the idea that the exoskeleton plays an important role in the heating process of beetles. The cuticle would be an excellent quality overcoat able to act passively in the thermal control of body temperature without implying energetic costs and metabolic changes. This may constitute a promising research avenue: it is necessary to estimate the degree of generality of this ability in Coleoptera, their phylogenetic signal along the evolution of the group, and their importance in explaining the capacity of these animals to overcome or be conditioned by the environmental changes.

##  Supplemental Information

10.7717/peerj.3349/supp-1 Supplemental Information 1 Rdata and Rscript R raw data and R script

## Additional Information and Declarations

### Data Availability

The following information was supplied regarding data availability: Both the raw data and R script are supplied as Supplemental Information.
